# Monitoring distal radius fractures during rehabilitation using infrared thermography

**DOI:** 10.3389/fphys.2026.1830281

**Published:** 2026-05-19

**Authors:** Manuel Rovira-Esteve, Eduardo Sánchez-Ramos-Cabellero, Mа Fe Miguez-Rey, Rosa M. Cibrian-Ortiz de Anda, Jose Ignacio Priego-Quesada

**Affiliations:** 1 Research Group in Sports Biomechanics (GIBD), Department of Physical Education and Sports, Universitat de València, Valencia, Spain; 2 Rehabilitation Unit, Manises Hospital, Manises, Spain; 3 Research Group in Medical Physics (GIFIME), Department of Physiology, Universitat de València, Valencia, Spain; 4 Traumatology and Orthopedic Surgery Department, Clinical Hospital of Valencia, Valencia, Spain

**Keywords:** asymmetries, bone injury, inflammation, recovery, thermal image

## Abstract

**Introduction:**

Distal radius fractures are among the most common injuries with a relevant rehabilitation process. Following a tissue injury, an inflammatory response occurs, leading to an increase in local temperature. This temperature can be recorded using infrared thermography. This study aimed to determine the degree of skin temperature asymmetry in patients with distal radius fractures during the rehabilitation process, at the beginning and at discharge, as well as to examine the correlation between thermal asymmetry and clinical indicators of injury.

**Methods:**

Infrared images of the forearms with distal radius fractures were taken from 17 patients at two time points: the first during the initial visit with the rehabilitation physician, and the second at the time of medical discharge. In addition, different clinical variables were recorded (e.g., pain perception, the degree of dorsal flexion, palmar flexion, supination, or pronation, etc.).

**Results:**

The injured wrist exhibited higher skin temperature than the non-injured wrist (mean difference: 0.8–2.0 °C; maximum: 0.9–1.8 °C; minimum: 0.5–2.1 °C) at the beginning of the rehabilitation. In the second visit, at the time of patient discharge, none of the thermal variables showed differences between the two extremities (p>0.05). Dorsiflexion was the only clinical variable that showed an association with thermal asymmetries (R^2^ = 0.10).

**Discussion:**

These findings support the use of infrared thermography as a complementary, non-invasive technique for monitoring inflammation and recovery during the rehabilitation of distal radius fractures.

## 1 Introduction

Bone fractures disrupt not only the structural integrity of the skeletal system but also initiate complex biological responses involving vascular injury and inflammation (Calmar and Vinci, 2002; McCarthy, 2006). The mechanical trauma causes damage to both macrobarriers (e.g., periosteum, cortical bone) and microbarriers (e.g., cellular membranes), leading to the release of damage-associated molecular patterns that signal immune activation (Tu and Li, 2023). These molecules are recognized by resident immune cells, triggering an inflammatory cascade that includes the release of vasoactive amines such as histamine (Thurmond et al., 2008). Histamine binds to H1 receptors in the endothelium, promoting nitric oxide synthesis and subsequent vasodilation (Thurmond et al., 2008; Tu and Li, 2023). Increased blood flow to the injured area raises the local temperature, which can be conducted to the skin via tissue conduction and blood convection (Hildebrandt et al., 2010; Xu and Werner, 1997). This temperature rise is often visible through infrared thermography, especially when compared with the contralateral, uninjured site (Escamilla-Galindo et al., 2025; Haluzan et al., 2015; Hildebrandt et al., 2010).

Infrared thermography is a non-invasive imaging technique that enables the measurement of skin temperature, and its use has expanded in recent decades for injury detection (Der Strasse et al., 2022; Fernández-Cuevas et al., 2017). Under normal conditions, the skin exhibits thermal symmetry (Niu et al., 2001; Vardasca et al., 2012). Vardasca et al. (2012) reported that the thermal difference between limbs typically does not exceed 0.5°C, with a standard deviation of 0.3°C. For this reason, the presence of thermal asymmetry (>0.5°C – 0.7°C) may indicate the existence of an injury or tissue alteration (Fernández-Cuevas et al., 2017; Niu et al., 2001; Vardasca et al., 2012). In addition to injury detection application, infrared thermography has been proposed as a tool for monitoring the progression of injuries until full recovery (Escamilla-Galindo et al., 2025; Jurić et al., 2024). However, the non-specific nature of thermal responses makes it difficult to determine the etiology of the injury (being proposed as a complementary technique) (Sillero-Quintana et al., 2015), and no clear relationship has been established between biochemical markers and skin temperature responses (De Carvalho et al., 2021; Silva et al., 2018). A meta-analyses by Sanchis-Sánchez et al. (2014) concluded that there is insufficient evidence to support the clinical utility of thermography. Nevertheless, the limited number of studies included in that review restricts the strength of its conclusions (Sanchis-Sánchez et al., 2014). More recent meta-analyses, such as that of García-de-la-Peña et al. (2024), support its potential clinical application, while emphasizing the need for further research, particularly focused on specific types of injuries.

Distal radius fractures are among the most common injuries and are increasing in incidence, with falls being the primary cause (Koo et al., 2013; Rundgren et al., 2020). Despite their prevalence, studies using infrared thermography in this context are scarce. One notable study by Haluzan et al. (2015) reported temperature asymmetries of 1.2 ± 0.5°C during the first-week post-injury, with the fractured forearm showing higher temperatures. The asymmetry peaked at week three (1.4 ± 0.5°C), gradually decreasing thereafter, with thermal symmetry restored by week 23 (0.2 ± 0.3°C). A similar study by Reed et al. (2020), focusing on children, assessed the effect of hand positioning during imaging. They found an asymmetry of 0.4 ± 0.6°C 24 hours after the fracture, regardless of hand position (flat or inclined). Although these studies offer preliminary reference values for thermographic analysis in distal radius fractures, further research with larger sample sizes is needed to strengthen the evidence supporting the use of infrared thermography for injury monitoring.

The aim of this study was to determine the degree of skin temperature asymmetry in patients with distal radius fractures during the rehabilitation process, at the beginning and at discharge, as well as to examine the correlation between thermal asymmetry and clinical indicators of injury. We hypothesized that skin temperature asymmetries would exceed 0.5 °C at the beginning of rehabilitation, decrease to below 0.5 °C by discharge, and show moderate correlations with clinical indicators of injury severity.

## 2 Methods

### 2.1 Study design

This observational descriptive study was conducted with a group of 17 patients diagnosed with distal radius fractures. Thermal images of both wrists were obtained at two time points: the first during the initial visit with the rehabilitation physician (after immobilization time), the second at the time of discharge from rehabilitation (upon completion of the rehabilitation program as determined by the treating physician).

### 2.2 Participants

The sample size was calculated using G*Power software (version 3.1.9.7, Universität Kiel, Germany) based on the effect size derived from the difference in mean skin temperature asymmetry between the initial visit and medical discharge in the first five participants, using a paired-samples Student’s t-test. An effect size of 0.8 was obtained, resulting in a required minimum sample size of 15 participants, assuming an α error of 0.05 and a statistical power of 90%. Then, seventeen patients (5 men and 12 women, aged 59 ± 19 years) referred from the Orthopedic Surgery and Traumatology Department of the Hospital Clínico Universitario de Valencia with a distal radius fracture participated in the study. They were informed about the study details through a phone call. Additionally, it was explained that their participation would not affect the standardized treatment prescribed by their physicians.

Due to potential inter-participant differences in skin temperature, a standardized protocol was established to minimize variability and enhance reliability and reproducibility. The following instructions were provided (Moreira et al., 2017): participants were instructed to avoid alcohol consumption, smoking, caffeine intake, heavy meals, or showering within 2 hours before the evaluation. Additionally, they were advised to avoid performing exercise and ointments/cosmetics on the skin the day of the measure, and to avoid sun exposure or UV radiation sessions 24 hours before the measurement.

To ensure the validity of the study and the homogeneity of the sample, specific inclusion criteria were established to select patients with characteristics related to the management of distal radius fractures. These criteria included: (1) patients who underwent surgical intervention for a distal radius fracture, allowing for the inclusion of cases treated with invasive procedures and conservative treatment; and (2) patients managed conservatively with closed cast or forearm splint immobilization following a distal radius fracture. Exclusion criteria were defined to eliminate factors that could interfere with the accuracy of thermographic measurements or bias the interpretation of results. These included: (1) participants requiring three or more additional medical visits during the rehabilitation process, which could indicate uncontrolled complications; (2) inability to follow the instructions provided; (3) presence of fever (>38°C, measured axillary), indicating systemic inflammation; and (4) intake of anti-inflammatory medications within three hours before the measurement, as these may reduce local inflammation and obscure thermal differences.

All patients provided written informed consent. The study was approved by the Ethics Committee of the University of Valencia and complied with the Declaration of Helsinki.

### 2.3 Acquisition and analysis of thermal images

Skin temperature was measured using a thermographic camera (FLIR E60bx, Flir Systems Inc., Oregon, USA), with an infrared resolution of 320x240 pixels and a thermal sensitivity of 0.05 °C. Measurements were conducted in a room with stable environmental conditions (23.8 ± 0.6 °C and 49 ± 3% relative humidity) and without external radiation sources (e.g., sunlight, heating). Furthermore, natural sunlight was avoided in the room to prevent interference with the measurements.

The imaging protocol followed the guidelines of the Thermographic Imaging in Sports and Exercise Medicine Checklist (Moreira et al., 2017) Participants underwent a 10-minute period to room temperature adaptation (Marins et al., 2014), during which they remained seated, with their forearms uncovered and avoiding contact with any surfaces. The camera was positioned perpendicular to the participant’s hands and at a distance of 2 meters. After the 10-minute adaptation, for thermal image acquisition, the patient stood upright with 30° shoulder flexion in the sagittal plane and full elbow and wrist extension and pronation.

**Figure 1:**
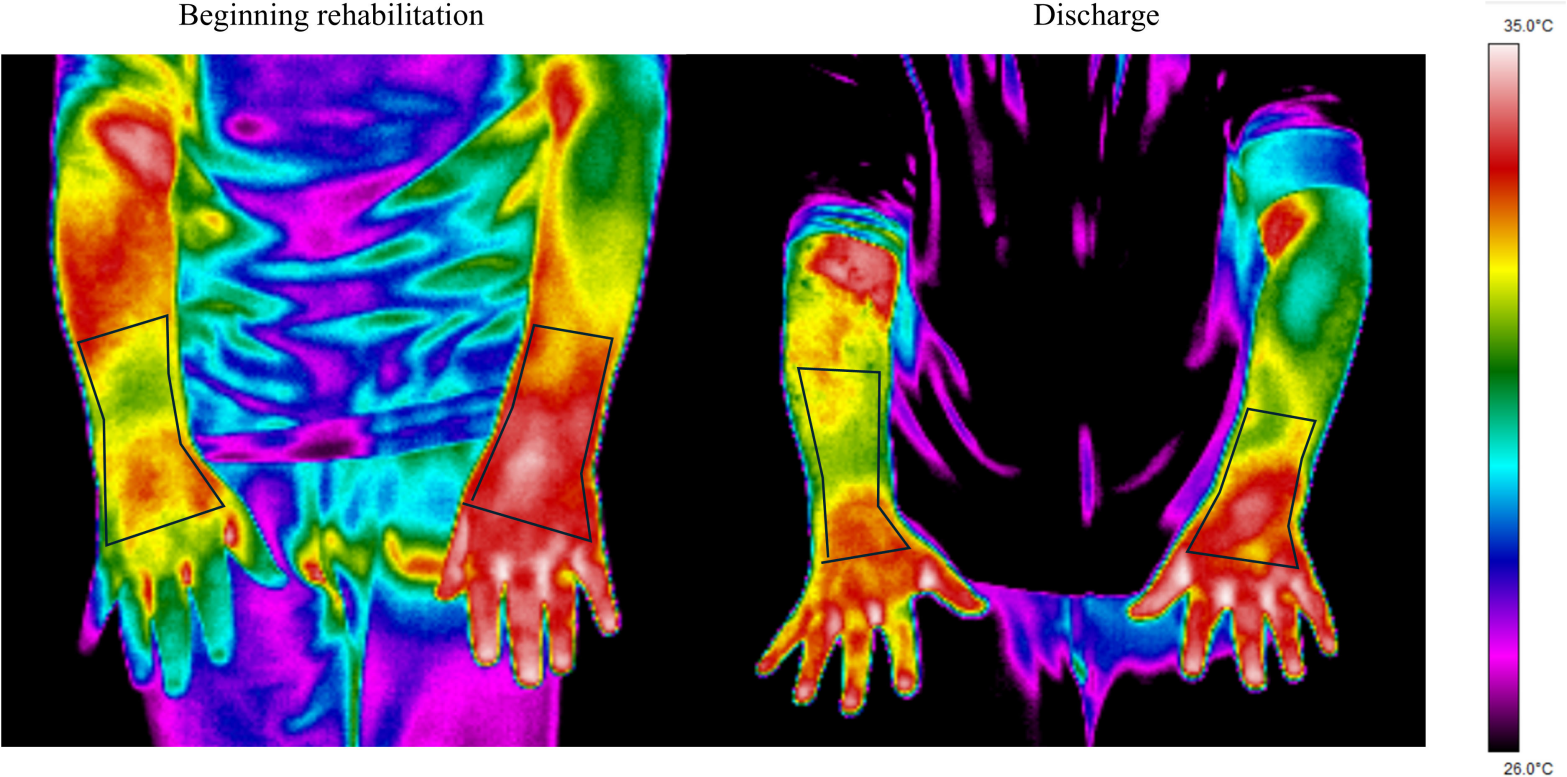
Region of interest determination. Example of one participant at the two measurement moments: beginning of the rehabilitation process and discharge.

The thermographic analysis of the forearm images was conducted using the ThermaCam Researcher Pro 2.10 software (Flir Systems Inc., Oregon, USA) by the same evaluator to minimize inter-observer bias. The region of interest (ROI) was defined as the area extending from the proximal third of the forearm to the bases of the metacarpal bones (**Figure 1**). Mean, maximum, and minimum temperatures from the ROI were obtained using an emissivity of 0.98 (Steketee, 1973). Moreover, asymmetries of the mean and maximum temperatures were calculated as the difference between the injured and non-injured ROIs.

### 2.4 Clinical variables

All participants were rehabilitated by the same physician. Discharge from rehabilitation was defined as the point at which the treating physician considered that the patient had achieved functional recovery, based on clinical criteria including pain resolution or minimal pain, recovery of wrist range of motion, and the ability to perform daily living activities without limitation.

The time elapsed from the injury to the measurement was recorded, as well as the time of immobilization before rehabilitation. The clinical variables registered in the two measurement moments were (Decarli et al., 2012; Lenoble et al., 1995): 1) whether the patient experienced pain or not, 2) the presence of paresthesia, 3) the degree of dorsal flexion, 4) the degree of palmar flexion, 5) the degree of supination, 6) the degree of pronation, 7) whether the patient could fully close their fist, 8) whether they could perform the pinching gesture and with which fingers, and 9) the opposition of the fingers according to the Kapandji classification (Kapandji, 2008).

### 2.5 Statistical analysis

The statistical analysis was conducted using RStudio software (version 2024.12.1). Normality was assessed for all variables using the Shapiro-Wilk test. In general, clinical variables and standard deviation of the ROI showed a non-normal distribution (p<0.05). The mean, maximum, and minimum temperatures, as well as the asymmetries of the mean and maximum temperatures, followed a normal distribution (p>0.05).

Differences in clinical variables between the start of rehabilitation and discharge were analyzed using the Wilcoxon test for non-parametric variables, the paired Student’s t-test for parametric variables, and the Chi-square test for categorical variables. For thermal variables, differences between the affected and healthy wrist at each measurement time were analyzed using the paired Student’s t-test, while the standard deviation of the ROI was assessed using the Wilcoxon test. Similar analyses were performed to examine differences between visits in the asymmetries of mean and maximum temperatures.

Additionally, multiple regressions with stepwise variable selection were conducted to analyze the relationship between baseline mean and maximum temperatures and clinical variables at both visits. Cohen’s effect size (d) was calculated for quantitative variables and classified as small (<0.5), moderate (0.5 - <0.8), and large (≥0.8). For categorical variables, Cramer’s effect size (v) was used and classified as small (<0.1), moderate (0.1 – 0.2), and large (≥0.2). Data are presented as means, standard deviations, and 95% confidence intervals (95% CI). Significance was established at p<0.05.

## 3 Results

### 3.1 Clinical variables

Of the 17 participants, 5 were treated conservatively and 12 underwent surgical intervention. The time of immobilization before the rehabilitation was 4 ± 2 weeks. The time that had passed from the injury to the measurement was 46 ± 16 days at the start of rehabilitation, and 109 ± 18 days at discharge. **Table 1** shows the results of the clinical variables at the beginning of rehabilitation and at medical discharge. Dorsal flexion and opposition in the Kapandji test improved at the medical discharge, and both variables exhibited a large effect size (ES = 1.0).

**Table 1:** Mean (standard deviation) of the quantitative clinical variables and the number of cases (% of cases relative to the total) of the categorical clinical variables at the start of rehabilitation and discharge.

Variable	Start of rehabilitation	Discharge	p-value	Effect size
Pain (N Yes)	9 (60%)	6 (40%)	0.30	0.1
Paresthesia (N Yes)	4 (27%)	2 (13%)	0.70	0.1
Dorsal flexion (°)	30 (14)	45 (15)	<0.01	1.0
Palmar flexion (°)	34 (13)	46 (17)	0.05	0.8
Supination (°)	67 (29)	83 (18)	0.09	0.7
Pronation (°)	85 (13)	87 (8)	0.80	0.2
Fully fist close (N No)	5 (33%)	2 (13%)	0.40	0.2
Pinching (N incomplete)	4 (27%)	0 (0%)	0.10	0.3
Opposition Kapandji (classification)	7.1 (2.1)	8.6 (0.7)	0.03	1.0

### 3.2 Skin temperature

**Figure 2:**
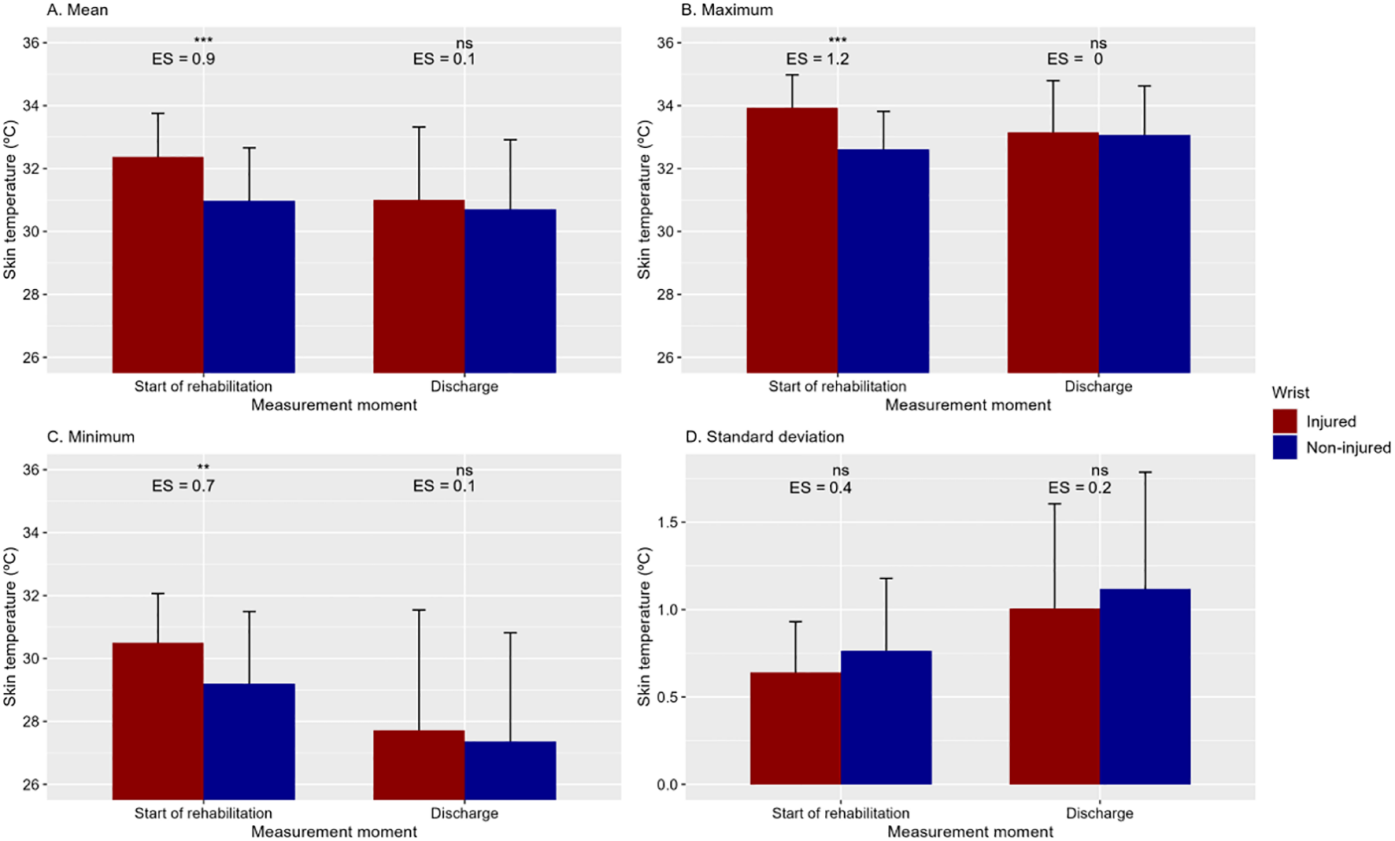
Mean and standard deviation of the mean, maximum, and minimum temperatures, as well as the standard deviation of the region of interest for the affected and healthy wrist in Visit 1 (beginning of rehabilitation) and Visit 2 (discharge). Differences between both extremities. The differences between both time points are presented using the p-value (ns no-significant; **p<0.01; ***p<0.001) and effect size (ES).

At the beginning of the rehabilitation, the affected wrist showed higher values than the healthy wrist in mean temperature [95%CI of the difference (0.8 – 2.0°C), large effect size], maximum temperature [95%CI (0.9 – 1.8 °C), large effect size] and minimum temperature [95% CI (0.5 – 2.1°C), moderate effect size] (**Figure 2**). The standard deviation of the ROI did not differ between both wrists at the start of rehabilitation. In the second visit, at the time of patient discharge, none of the variables showed differences between the two extremities.

**Figure 3:**
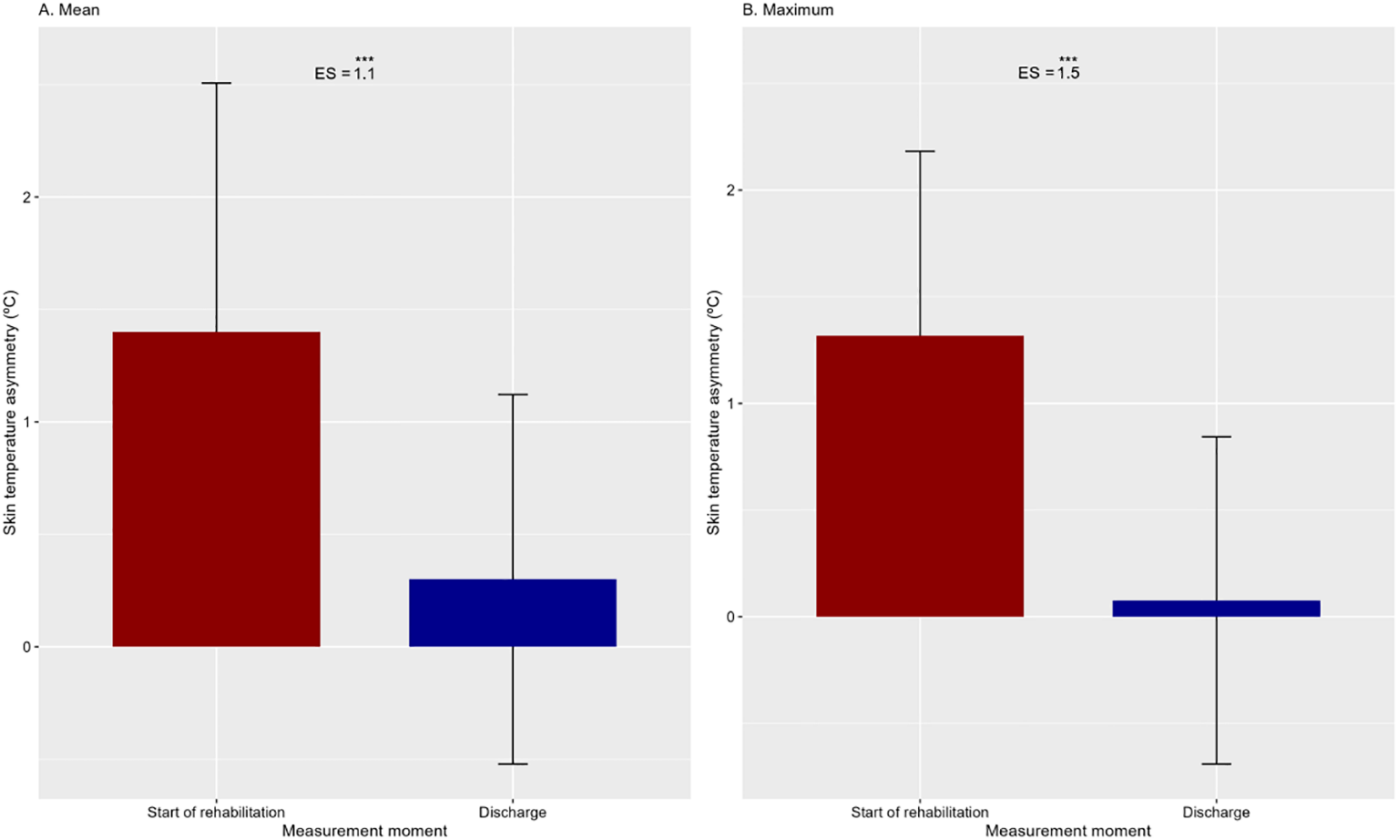
Mean and standard deviation of the asymmetry in average and maximum temperature at visit 1 (beginning of rehabilitation) and visit 2 (discharge). The differences between both time points are presented using the p-value (***p<0.001) and effect size (ES).

These findings were confirmed by the analysis of asymmetry in mean and maximum temperature (**Figure 3**), both of which were higher at the beginning of rehabilitation compared to discharge, with a large effect size [mean temperature asymmetry: 95% CI (0.5 – 1.7 °C); maximum temperature asymmetry: 95% CI (0.7 – 1.8 °C)].

The distribution of asymmetries in the analyzed sample is presented using a percentile table (**Table 2**). It can be observed that 75% of the sample presented an asymmetry in the mean and maximum temperature greater than 0.7 °C at the beginning of rehabilitation, and at discharge, this percentage was only 25% for the mean temperature, and 10% for the maximum temperature.

**Table 2:** Percentile distribution of the asymmetry in mean temperature and maximum temperature.

Percentile	Mean skin temperature asymmetry (°C)		Maximum skin temperature asymmetry (°C)	
	Start of rehabilitation	Discharge	Start of rehabilitation	Discharge
10	0.07	-0.64	0.47	-0.62
25	0.73	-0.15	0.68	-0.35
50	1.25	0.30	1.10	0.20
75	1.55	0.65	1.63	0.40
90	3.23	1.23	2.43	0.78

### 3.3 Relationship between clinical variables and thermal asymmetries

For both mean and maximum asymmetry, the stepwise variables selection method for multiple regressions identified dorsal flexion as the only variable that showed an inverse relationship with both thermal variables (**Table 3**). However, these association explained a low percentage of the variance (10% for the mean skin temperature and 24% for the maximum skin temperature).

**Table 3:** Regressions were obtained between skin temperature asymmetries and dorsal flexion.

Variable	Mean skin temperature asymmetry (°C)		
	Coefficient	Standard error	R^2^ (p-value) of the model
Intercept	1.66	0.41	0.10 (p=0.04)
Dorsal flexion	-0.02	0.01	
	Maximum skin temperature asymmetry (°C)		
	Coefficient	Standard error	R^2^ (p-value) of the model
Intercept	1.75	0.35	0.24 (p<0.01)
Dorsal flexion	-0.03	0.01	

## 4 Discussion

The objective of this study was to determine the degree of skin temperature asymmetry in distal radius fractures at the beginning of rehabilitation and at medical discharge, and to relate these asymmetries to clinical indicators of injury. The main findings showed that at the start of rehabilitation, after 46 days post-injury and 4 weeks of immobilization, the injured wrist exhibited higher skin temperature than the non-injured wrist (mean difference: 0.8–2.0 °C; maximum: 0.9–1.8 °C; minimum: 0.5–2.1 °C). At discharge, no significant temperature differences were observed between the wrists. Additionally, dorsiflexion was reduced at the start of rehabilitation and improved by discharge. This variable was the only one significantly associated with thermal asymmetries, explaining between 10% and 24% of their variance.

At the beginning of rehabilitation, the mean skin temperature of the affected wrist was consistently higher than that of the healthy wrist across all parameters: mean, maximum, and minimum values. Specifically, the mean temperature difference ranged from 0.8 °C to 2.0 °C. These results are in line with previous studies reporting thermal asymmetries in bone fractures (Der Strasse et al., 2022; Haluzan et al., 2015; Reed et al., 2020; Sillero-Quintana et al., 2015). For instance, Haluzan et al. (2015) observed that during the first week after the fracture, the fractured wrist exhibited a temperature increase of 0.7 to 1.7 °C compared to the contralateral side. Similarly, Reed et al. (2020) found a mean asymmetry of 0.5 ± 1.8 °C. Der Strasse et al. (2022) also reported a 1.0 °C temperature difference between fractured and non-fractured forearms one day later. Differences between studies could be related to the measurement time after the injury. Given that thermal asymmetry reflects inflammatory activity and vasodilation, the nature and severity of the injury likely influence its magnitude. Reed et al. (2020) showed that although both fractures and sprains elevated skin temperature, fractures produced significantly greater increases. Together, their findings and ours support the potential of infrared thermography to help distinguish between different types of injuries.

By the end of rehabilitation, thermal asymmetries had diminished, resulting in thermal symmetry between wrists. Tissue injury causes inflammation and vasodilation, leading to an increase in skin temperature (Hildebrandt et al., 2010). Therefore, the observed temperature decrease likely reflects reduced inflammation (Escamilla-Galindo et al., 2025). This supports the use of infrared thermography as a non-invasive tool for monitoring inflammatory status and evaluating the effectiveness of the rehabilitation process, consistent with findings from studies on anterior cruciate ligament rehabilitation (Escamilla-Galindo et al., 2025).

Among the clinical variables measured, both the Kapandji opposition test and dorsiflexion improved during the rehabilitation period. However, only dorsiflexion showed a statistically significant inverse relationship with maximum and minimum thermal asymmetries. Although an interpretation of this association is provided below, it is important to note that it explains only 10–24% of the variance and should therefore be interpreted with caution. A possible explanation lies in wrist anatomy. According to Kapandji (2008), the distal radius epiphysis, prone to fracture from extension-related trauma, serves as an attachment site for key wrist and finger flexor tendons. These include the flexor digitorum profundus, flexor digitorum superficialis, flexor carpi radialis, and palmaris longus. Trauma in this region may impair their function, especially in the capacity for passive stretching and active contraction (Almekinders, 1999). As inflammation subsides, tendon mobility and muscle function may improve, which could explain the relationship with dorsiflexion (Järvinen et al., 2005; Souza and Gottfried, 2013). In this regard, studies such as that of Liddy et al. (2026) observed that after surgery following a distal radius fracture, the average active wrist flexion was 52.7°, considering that the normal range of flexion is up to 85° (Kapandji, 2008). In the study by Lenoble et al. (1995), this flexion, after surgery using the Kapandji method, averaged 59°. In contrast, other clinical variables such as pronation and supination were not significantly associated with thermal asymmetries. One possible explanation is related to the review by Almekinders (1999): the affected region results in a reduction of biomechanical capacity. However, it can be assumed that the supinator and pronator muscles, being located around the humerus and the proximal area of the radius and ulna, are not affected. This is reflected in the previously mentioned study, where after surgery using the Kapandji method, the supination and pronation angles were 76° and 80°, respectively, with the normal range of supination reaching up to 90° and pronation up to 85° (Kapandji, 2008).

This study has several limitations. Because data collection was limited to two time points (start and end of rehabilitation), additional measurements, particularly closer to the injury event and at mid-rehabilitation, could have provided a more detailed thermal profile. This would have been interesting because the response to treatment is known to be diverse (Quadlbauer et al., 2020), as shown by the data from the present study, in which the standard deviation of rehabilitation time was 18 days. Moreover, incorporating biochemical markers of inflammation or diagnostic imaging would have allowed for deeper correlations with thermographic data. Another limitation of this study is the inclusion of both conservatively and surgically treated patients without performing a subgroup analysis. Due to the small and unbalanced sample (only five patients received conservative treatment), such comparisons were not statistically feasible. Differences in healing processes, immobilization duration, and the potential influence of surgical materials may affect thermographic responses and should be explored in future studies with larger and more homogeneous samples. Future research is needed to establish reference values for thermal asymmetry at different stages of rehabilitation and to evaluate its utility in distinguishing between injury types (e.g., fracture vs. sprain).

## 5 Conclusion

Distal radius fractures result in significant thermal asymmetry in the affected wrist compared to the non-injured side. This asymmetry progressively decreased throughout the rehabilitation process, culminating in thermal symmetry at discharge. Dorsiflexion was the main clinical variable inversely associated with thermal asymmetry. These findings support the use of infrared thermography as a complementary, non-invasive technique for monitoring inflammation and recovery during the rehabilitation of distal radius fractures.

## Data Availability

The raw data supporting the conclusions of this article will be made available by the authors, without undue reservation.
